# Nuclear incorporation of iron during the eukaryotic cell cycle

**DOI:** 10.1107/S1600577516012807

**Published:** 2016-10-18

**Authors:** Ian Robinson, Yang Yang, Fucai Zhang, Christophe Lynch, Mohammed Yusuf, Peter Cloetens

**Affiliations:** a Research Complex at Harwell, Rutherford Appleton Laboratory, Didcot, Oxon OX11 0FA, UK; b London Centre for Nanotechnology, University College London, London WC1E 6BT, UK; c Condensed Matter Physics and Materials Science Department, Brookhaven National Laboratory, Upton, NY 11973, USA; d ESRF – The European Synchrotron, 71 Avenue des Martyrs, 38000 Grenoble, France

**Keywords:** fluorecence, beamline, chromosome, imaging, phase contrast

## Abstract

Scanning X-ray fluorescence microscopy has been used to probe the distribution of S, P and Fe within cell nuclei. Nuclei, which may have originated at different phases of the cell cycle, are found to show very different levels of Fe present with a strongly inhomogeneous distribution. P and S signals, presumably from DNA and associated nucleosomes, are high and relatively uniform across all the nuclei; these agree with X-ray phase contrast projection microscopy images of the same samples. Possible reasons for the Fe incorporation are discussed.

## Abstract 2

Scanning X-ray fluorescence microscopy has been used to probe the distribution of S, P and Fe within cell nuclei at the new ID16 beamline. Estimates of P, S and mass signals for the chromosomal matter agree with quantitative X-ray phase contrast projection microscopy images of the same samples, while fluorescence shows Fe incorporation.

## 1. Introduction   

The eukaryotic cell nucleus contains all the genetic material responsible for the propagation of life from one cell generation to the next. All the cell’s DNA resides there, confined within a porous nuclear envelope, along with over a hundred different protein molecules (Uchiyama *et al.*, 2005 ▸) associated with chromatin, the DNA-protein complex making up the chromosomes. Over the course of the cell cycle, the mass of DNA increases from two copies to four during S phase, then returns to two copies upon cell division during metaphase (M-phase). The DNA mass remains constant during the two intervening growth phases G1 and G2. For human cells, investigated here, the DNA mass should correspond to the length of the human genome, which is 3.2 × 10^9^ base pairs. Quantitative assessment of the amounts of DNA and protein can be made by X-ray fluorescence using the P signal for DNA and the S signal for protein, noting that more than two-thirds of the nuclear protein is in the form of histones, which bind stoichiometrically to DNA through the nucleosomes, and which contain a known amount of cysteine and methionine (Uchiyama *et al.*, 2005 ▸).

X-ray imaging of biological materials has two important advantages over electron microscopy: (i) the X-ray penetration, sufficient to avoid sectioning of the samples, and (ii) its excellent chemical sensitivity for elemental analysis using fluorescence. In this work, the new Nano-Imaging beamline ID16A-NI, part of the UPBL04 ‘NINA facility’ built in the framework of the ESRF Upgrade Programme (Pacureanu *et al.*, 2016 ▸), was used to image human cell nuclei. The size of a human cell nucleus is in the range of 10 µm, which falls within the field of view of the propagation-based phase contrast imaging capability of ID16A. In addition, high-resolution substructure is expected, at least if the nuclei are close to the metaphase point of the cell cycle when the parent cell is preparing for division. For the X-ray fluorescence imaging capabilities of ID16A, known quantities of DNA and (to a slightly lesser extent) proteins are expected to be present in a cell nucleus, which can be used in quantitative chemical analysis and to verify the calibration of the sensitivity.

Nuclei close to metaphase were targeted in this study because of interest in the higher-order structure of the separated chromosomes located within them, but it was also appreciated that this really needs a three-dimensional imaging capability to segregate them. Our sample preparation methods make use of cell cycle checkpoint inhibitors to synchronize the cells during culture, but this still allows some nuclei to emerge from the preparations at other points of the cell cycle. Centrifugation is used to remove cytoplasm and most of the other cell components (Yusuf *et al.*, 2014 ▸), so a relatively pure preparation of whole nuclei and individual chromosomes is obtained, many with the nuclear membrane intact. This strictly excludes nuclei in late metaphase, once the nuclear membrane dissolves, but does include prophase just beforehand, when the 46 chromosomes are fully condensed within a nucleus. If the cells were in G1 phase when the samples were prepared, they would contain two double-stranded copies of all the genomic DNA; if they were in G2 phase or the beginning of metaphase (M phase), there should be four copies; in S phase, there would be somewhere between two and four copies.

The full human genome contains 3.2 × 10^9^ base pairs per double-strand of DNA, which is divided into the 23 chromosomes. Associated with each base pair are two phosphates, one on each strand. These are the largest expected contribution to the P X-ray fluorescence signal, with small additional amounts coming from buffers, the lipids in the cell membranes and any residual RNA or ATP. So a cell nucleus should have a well defined signal from these 2.6 × 10^10^ P atoms in its fluorescent images if it is in the second half of the cell cycle (G2 or M phase), or 1.3 × 10^10^ P atoms in its fluorescent images if it is in the first half of the cell cycle (G1 phase).

Similarly, the S X-ray fluorescence signal would be mostly attributed to cysteine (Cys) and methionine (Met) residues in the nuclear proteins. Fortunately, much is known about the make-up of the (mostly structural) chromosomal proteins found in metaphase from the work of Uchiyama *et al.* (2005 ▸): 71% of the total mass is histones, which are the core proteins around which the DNA is spooled to make nucleosomes. The histones contain many basic arginine and lysine groups, which help neutralize the negative charge carried by the DNA. One nucleosome typically occupies 170 base pairs of DNA and, since most of the DNA can be assumed to have condensed into nucleosomes, we can use this to estimate the expected total amount of protein per nucleus. Moreover, the histone sequences are all known, so we can expect there to be 14 S atoms (2 × Cys and 12 × Met) per 170 base pairs of DNA associated with the histones (Mariño-Ramírez *et al.*, 2011 ▸). We therefore expect a cell nucleus to have 2.1 × 10^9^ S atoms in its X-ray fluorescence images in G2 or M phase and 1.0 × 10^9^ S atoms in G1 phase.

The total mass of DNA and protein expected in metaphase can also be estimated from the size of the human genome. This can be compared with quantitative masses measured by X-ray phase contrast imaging. One base pair weighs 650 Da, so one double-stranded copy of 3.2 × 10^9^ base pairs weighs 3.5 pg. A nucleosome octamer, the core protein of a single nucleosome, weighs 110 kDa. So with one nucleosome attached to every 170 base pairs we expect 3.5 pg of histones; allowing for 29% of non-histone protein brings this estimate to 4.9 pg per double-stranded genome copy. In the first half of the cell cycle (G1), we expect to find 16.8 pg of DNA and protein directly associated with the chromosomes. In the second half of the cell cycle (G2/M), we expect 33.6 pg.

The presence of Fe in the cell nucleus has been discussed repeatedly in the scientific literature. Yagi *et al.* (1992 ▸) have suggested there may be an evolutionary connection between iron and DNA because of the powerful redox potential of Fe. Fe is an essential element of proteins, often in the form of iron–sulfur (FeS) clusters used in electron transport enzymes (Johnson *et al.*, 2005 ▸) or in heme complexes in cytochromes (Dawson, 1988 ▸). Iron can be toxic to cells *via* the generation of free radicals (Yagi *et al.*, 1992 ▸). Since the presence of iron can lead to DNA damage pathways, there may be evolutionary advantage to keeping the DNA in its own nuclear compartment, away from many of the metabolic processes.

Despite the view that Fe-containing enzymes would not be widely used within the nuclear compartment of the cell, there have been recent reports of FeS-containing enzymes directly involved with DNA replication. DNA primase was found to contain an FeS domain (Klinge *et al.*, 2007 ▸; Weiner *et al.*, 2007 ▸) along with DNA helicase (Wu & Brosh, 2012 ▸) and DNA repair glycosyl­ases (Wu & Brosh, 2012 ▸). A review by Lill *et al.* (2006 ▸) named five associations of FeS proteins with the cell nucleus: DNA glycosyl­ase (Ntg2), histone acetyl­transferase (Elp3), P-loop ATPase (Nbp35), iron-only hydrogenase (Nar1) and ABC protein (Rli1). All of these functions are believed to be associated with DNA replication and repair so should be expressed only during S phase of the cell cycle and should be absent during other phases.

Ferritin, the eukaryotic iron storage protein, is not expected to be co-localized with DNA, yet this was reported in a few diverse examples by Thompson *et al.* (2002 ▸). Nuclear ferritin might be associated with the protection of DNA or conversely with oxidative DNA damage. If nuclear ferritin is present, it might be expected to be associated with the nuclear membrane, rather than mixed in with the DNA-containing chromatin.

One organelle little discussed in relation to iron transport and accumulation is the nucleolus, a subcompartment of the nucleus which appears at certain points of the cell cycle. There are very few mentions in the literature of nucleolar iron. It was recently shown that plant nucleoli contain iron (Roschzttardtz *et al.*, 2011 ▸), purportedly bound to ribosomal RNA (rRNA). It was suggested that iron might stabilize secondary RNA structures in the nucleolus or otherwise catalyse maturation of rRNA subunits. The following year it was shown that human neuronal cells also contain nucleolar iron (Sukhorukovaa *et al.*, 2013 ▸), although no explanation was provided. It should be noted that the presence of nucleolar iron in HeLa cells was reported some decades ago (Robbins *et al.*, 1972 ▸). In the case of HeLa cells, it was clear that iron bound to proteins in the nucleolus and it was suggested that the organelle could be a Fe repository for iron-dependent DNA synthesis proteins. Whether or not the iron content of the nucleolus changes as a function of cell cycle phase remains to be determined.

To address these questions, synchrotron-based scanning X-ray fluorescence (SXRF) and phase contrast projection microscopy imaging of human cell nuclei with sub-cellular resolution were undertaken in the work reported here.

## 2. Methods   

### 2.1. Sample preparation   

Nuclei were prepared according to a previously published filtration-based protocol for chromosomes (Yusuf *et al.*, 2014 ▸) with modifications to preserve the intact nuclei. Human lymphocyte cells (GM18507) were cultivated at 37°C in a 5% CO_2_ incubator. The RPMI-1640 medium (Sigma Aldrich, UK) contained 20% fetal bovine serum (Sigma Aldrich, UK) and 1% l-glutamine. The cells were treated with colcemid (0.2 µgml^−1^, Gibco BRL) to arrest them in metaphase and were fixed in 3:1 methanol:acetic acid after 0.075 *M* KCl treatment. Following extraction, the nuclei were prepared for X-ray imaging as described by Shemilt *et al.* (2015 ▸). Samples were fixed in a buffer containing 0.5% glutaraldehyde, 10 m*M* HEPES-KOH and 5 m*M* MgCl_2_. The samples were pipetted in 2 µL drops onto 200 nm-thick silicon nitride membrane windows and stained with 150 µ*M* Sybr gold dye for optical fluorescence imaging. After washing in water, the samples were left to dry in air. They were imaged using a Zeiss AxioZ2 microscope (using *Metafer Isis* software) to obtain visible light and optical fluorescence images for reference and correlation with the X-ray results.

For X-ray imaging, several silicon nitride membranes were prepared with the same sample material. The resulting samples were found to contain a large number of intact nuclei, but also chromosome spreads and individual chromosomes from burst nuclei. Some of the membrane-bound samples were stained with platinum blue (Wanner & Formanek, 1995 ▸), at 5 m*M* for 30 min and washed in water. No significant differences were found in the X-ray phase contrast images; however, the Pt X-ray fluorescence *M*-line-signal was found to strongly interfere with the fitting of the X-ray fluorescence spectra due to overlap with the P *K*-lines. Results are therefore reported from samples prepared without Pt staining. After the X-ray experiment, the samples were reimaged with an Olympus LEXT 4000 confocal microscope to obtain further reference images of the relevant samples.

### 2.2. X-ray measurements   

The measurements were performed under vacuum (around 1 × 10^−7^ mbar) at room temperature on the new nano end-station of ID16A. The silicon nitride membrane windows were clamped into the insertion stubs designed for the sample stage of ID16A. Samples were transferred onto a piezo-driven short-range hexapod stage. The hexapod movement, under the control of capacitive sensors, was used to monitor the contact forces during sample changing.

The ID16A beamline has two multilayer coated Kirk­patrick–Baez (KB) focusing mirror pairs located at 185 m from an undulator source operating at 17 keV or 33.6 keV (Morawe *et al.*, 2015 ▸). The energy of 17 keV was chosen for best excitation of the X-ray fluorescence signal of all relevant elements. The KB system, with extreme demagnification designed for a 15 nm × 15 nm focus, produced a measured focus of 25 nm (H) × 37 nm (V) with a very high flux of 3.4 × 10^11^ photons s^−1^ from the broad bandpass (1%) of the multilayer.

To make correlative imaging of both morphology and elemental content in the same sample, phase contrast projection microscopy and X-ray fluorescence microscopy were combined in a sequence.

For morphological measurements, phase contrast images of the samples were firstly obtained by moving them downstream of the focus and recording Fresnel projection images at four distances. These distances between the focus and the sample were fixed to obtain a magnification yielding a 10 nm or 5 nm equivalent pixel size at the level of the sample. A FReLoN CCD-based indirect detector with 2048 × 2048 pixels was used to record the magnified projections, whose visible-light optics gave an effective detector pixel size of 0.84 µm. Seventeen projections for different lateral positions of the object were recorded, each taking 0.3 s exposure time and then averaged to obtain high-quality Fresnel projection images. Averaging is done to reduce the effect of inhomogeneities in the incoming beam, mainly related to the KB focusing optics, and to increase the signal-to-noise ratio. A full-field quantitative phase map was then retrieved from the four Fresnel diffraction patterns based on a contrast transfer function approach (Cloetens *et al.*, 1999 ▸). The phase map is proportional to a projection of the real part of the refractive index or the electron density in the specimen. As the sample consists mostly of light elements, the phase map is to a good approximation proportional to the projection of the mass density. Therefore all the projection phase maps were converted to areal density (µg mm^−2^). Combined with SXRF images, they can be used for normalization to yield true elemental average mass fractions of specific elements (Kosior *et al.*, 2012 ▸).

For SXRF measurements, the same sample was moved back to the focus position and scanned continuously across the beam with an equivalent step size of 30 nm and a dwell time of 50 ms. The X-ray fluorescence emission was collected on-the-fly by a pair of six-element silicon drift detectors (Sensortech, UK) positioned perpendicular to the beam path at each side of the sample. The freely available software *PyMca* (Solé *et al.*, 2007 ▸) was used for the analysis of the X-ray fluorescence spectra. At every scan point, the summed spectrum collected from the 12 detector elements was fitted to decompose it into the emission lines of the individual elements (*K*-emission lines for P, S and Fe). The absolute calibration to the elemental areal density (ng mm^−2^) was determined by fitting the fluorescence signal from a thin film standard (AXO Dresden GmbH).

## 3. Results and discussion   

**Figure 1:**
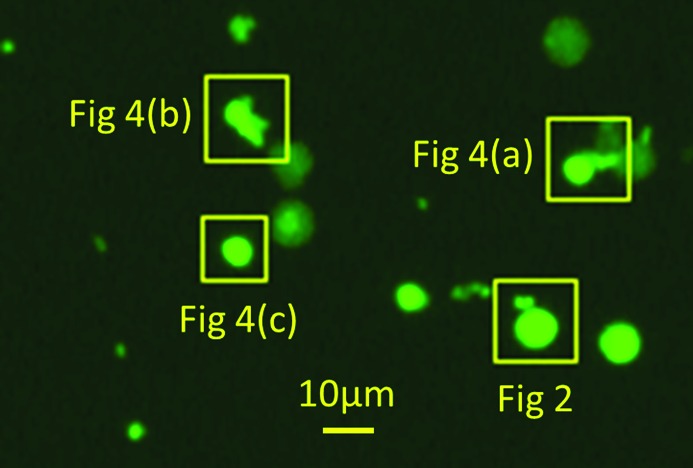
Low-magnification optical fluorescence image taken under the excitation conditions for Sybr gold dye using a Zeiss AxioZ2 microscope. Boxes and labels indicate the nuclei that are analysed further in this work.

Fig. 1 ▸ shows an overview optical fluorescence image taken under the excitation conditions for Sybr gold dye, which binds specifically to DNA. While the nuclei are clearly well isolated on the membrane, it is clear that not all of them are equally bright. This suggests that either the dye is unable to penetrate the samples uniformly or, more likely, that some nuclei have become depleted in their DNA content. This might have occurred during the washing steps of the sample preparation, or possibly during handling of the samples. We note that the image was taken shortly after sample preparation, before transporting the samples to ESRF, so this does not take into account the effects of the vacuum sample transfer into the ID16A instrument.

**Figure 2:**
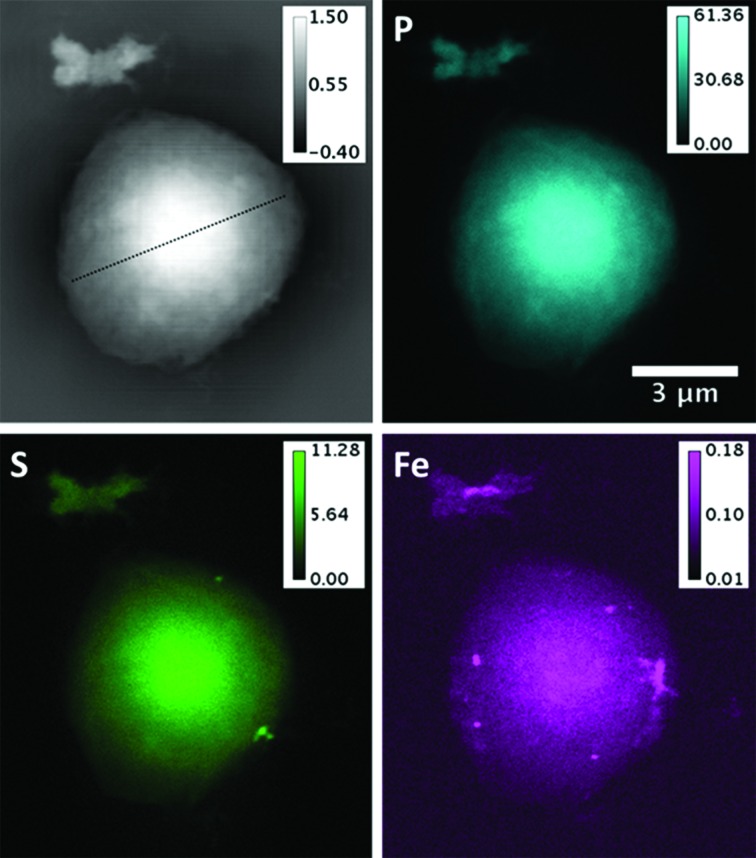
X-ray images of the nucleus outlined in Fig. 1 ▸, with a group of individual chromosomes on the upper side. Top left: phase contrast image presented as total areal density (unit: µg mm^−2^). Other panels: elemental areal density distributions from scanning X-ray fluorescence (unit: ng mm^−2^).

**Figure 3:**
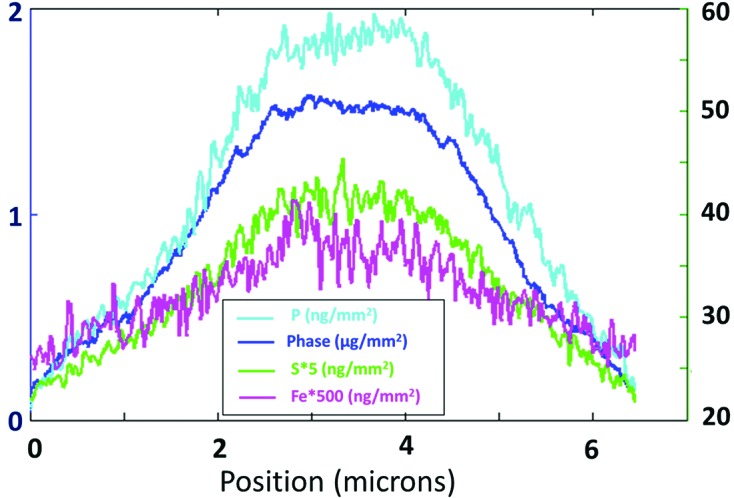
Line profiles across the nucleus shown in Fig. 2 ▸ showing the similarity of the shapes of the dome-shaped distributions in the phase map and the elemental maps.

Fig. 2 ▸ shows comparison images of an isolated nucleus by both available X-ray imaging methods: phase contrast projection microscopy and scanning X-ray fluorescence imaging of the P, S and Fe *K*-lines. The total signals for the three elements, integrated over the nuclear surface and calibrated in units of numbers of atoms, are listed in Table 1 ▸, along with the integrated mass. The field of view of this image also contains one or two individual chromosomes in a small cluster at the upper side. This nucleus contains the least quantity of Fe observed. The distributions of the P- and S-signals overlay well on top of each other and also agree with the distribution of the areal density seen in the phase map. The agreement in the spatial distributions of mass and the S, P and Fe concentrations can be clearly seen in the cross-sectional plot of Fig. 3 ▸. The dome-shaped distribution of all three images is roughly what would be expected for a spherical or hemispherical nucleus with a uniform density of chromatin matter within its volume.

The total P signal is estimated to come from 1.8 × 10^10^ ± 0.1 × 10^10^ P atoms, falling right in between the expected values for a nucleus in the first and second half of the cell cycle, 1.3 × 10^10^ and 2.6 × 10^10^ P atoms, respectively. This number suggests a small contribution from other sources, lipids, ATP, RNA or phosphate buffer, unless there is a calibration error. It is noteworthy that P has not substantially been lost during the sample preparation and insertion into vacuum. We did not detect any effect of radiation damage because the signal levels in the images were found to be reproducible upon repeated scanning. The total X-ray fluorescence S signal of 2.6 × 10^9^ ± 0.2 × 10^9^ atoms is found to be 24% higher than the estimate given above of 2.1 × 10^9^ S atoms in G2 or M phase. Since this appears to be homogeneously distributed within the chromatin-filled region of the nucleus, this suggests that the extra signal may be coming from the 29% non-histone proteins (Uchiyama *et al.*, 2005 ▸). We note, however, that histones tend to have relatively low levels of Cys and Met amino acids, and this may or may not apply to the related non-histone protein complement. We are also disregarding the contributions from non-chromatin proteins or microtubules associated with the nucleus at certain points of the cell cycle.

**Figure 4:**
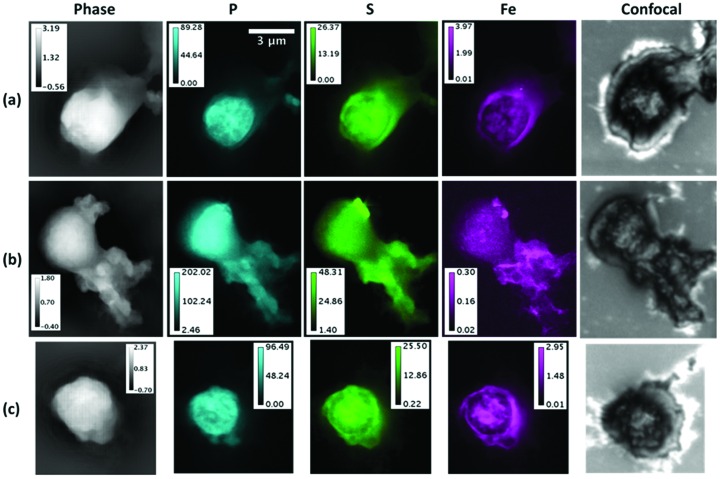
X-ray images of three more nuclei outlined in Fig. 1 ▸. Left column: phase contrast images presented as total areal density (unit: µg mm^−2^). Centre columns: elemental areal density distributions from scanning X-ray fluorescence (unit: ng mm^−2^). Right: optical confocal height map, measured after the X-ray experiment. The scale bar applies to all panels.

**Figure 5:**
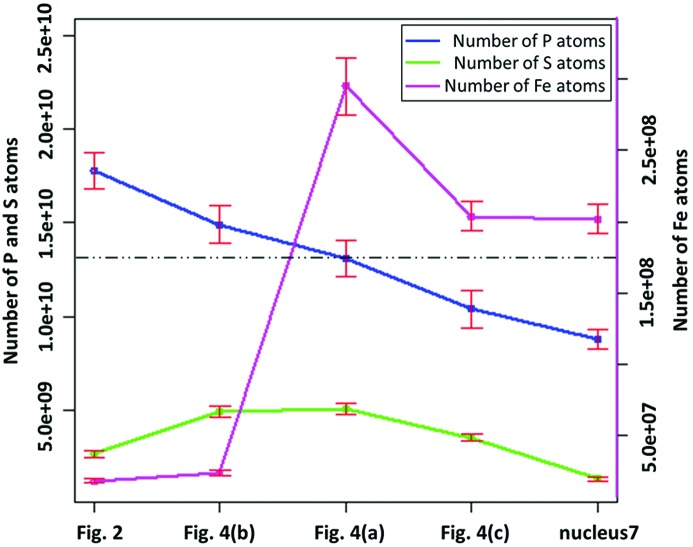
Variations of the numbers of atoms of P, S, and Fe of the five scanned human nuclei (listed in Table 1 ▸) with the error bars. *X*-axis: the five scanned nuclei. *Y*-axis (left): number of P and S atoms; *Y*-axis (right): number of Fe atoms. Dashed black line: expected number of P atoms of a nucleus in G1 phase.

Corresponding images from three more nuclei, as labelled in Fig. 1 ▸ and shown in Fig. 4 ▸, gave the integrated signals listed in Table 1 ▸ and Fig. 5 ▸. The average P content is close to that expected for a nucleus in the first half of the cell cycle G1. However, the S content is 1.7 times higher than the higher estimate for a nucleus in the G2 phase. For the P signals from the five nuclei measured, there are factors-of-two variations from one nucleus to another, which might indicate the level of inherent measurement errors or variations of sample preparation, but it may also indicate that the nuclei are captured at different points of the cell cycle. The variation of S signals is substantially greater, which suggests less of an effect of sample preparation and more likely indicating different levels of protein in the five nuclei associated with their stage in the cell cycle.

The observed nuclear masses also agree well with the estimates above for genomic DNA and chromosomal protein, with all values falling within the factor-of-two range expected for early/late points of the cell cycle. It is notable that the higher mass nuclei are also the ones showing high S signals, further supporting the suggestion that extra protein may be present in those nuclei.

**Figure 6:**
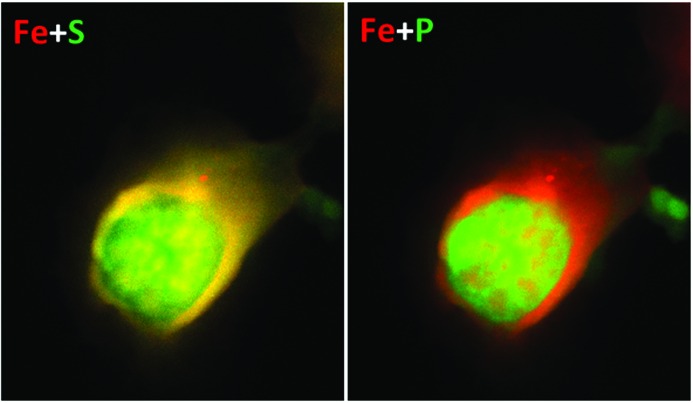
Nucleus in Fig. 4(*a*) ▸ shown as an overlay of the elemental maps of Fe and, respectively, S (left) and P (right). The yellow colour on the left indicates co-localization of Fe and S, while in the overlay of Fe and P we see more red and green colours.

Much greater variation was found in both the masses and distributions of the Fe signal, for which a 15× variation was found. Fig. 2 ▸ shows the nucleus with the smallest level of Fe, while that of Fig. 4(*a*) ▸ has the highest level. Unlike S and P, the Fe signals are strongly clustered and often seen to be located at the periphery of the nucleus. This is much better seen in the elemental overlay plots of Fig. 6 ▸. It is therefore concluded that most of the Fe signal is coming from the nuclear membrane structures rather than the central regions, as discussed further below.

We also note that the separated chromosome structure seen at the top of Fig. 2 ▸ has co-localized P and S signals coming from its distinct arm regions and a separate Fe signal in the centre, which is depleted in P and S. This appears to be an agglomeration of two chromosomes on the left and right sides and may contain a piece of Fe-rich nuclear membrane in the centre.

**Figure 7:**
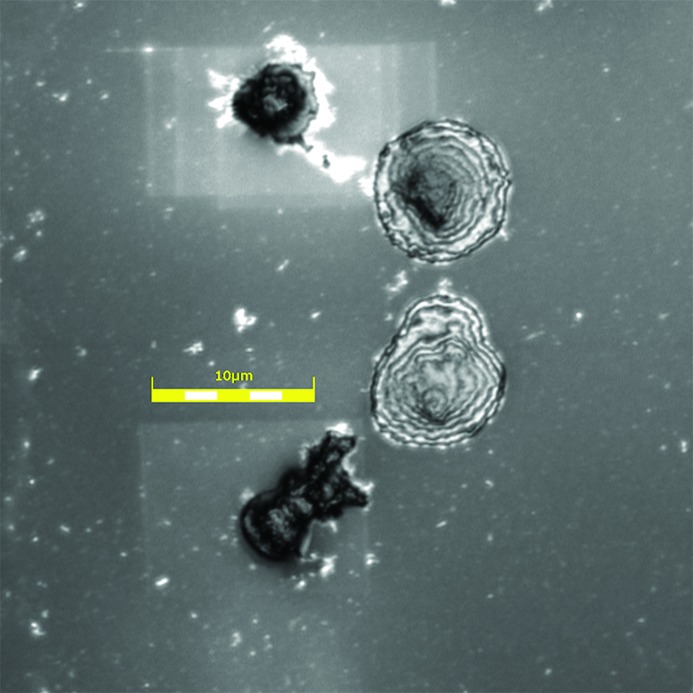
Grey-scale confocal image measured with a 50× lens on an Olympus LEXT 4000 microscope after the X-ray experiment. Nuclei 4(*b*) and 4(*c*) can be seen, along with a clear modification to the membrane in the region where the fluorescence mapping had taken place.

Finally, in Fig. 7 ▸, we include the result of optical confocal microscope imaging of the membranes after removal from the beamline, showing a larger area surrounding the nuclei of Figs. 4(*b*) and 4(*c*) ▸. Confocal images of each of the measured nuclei in Fig. 4 ▸ are included in the right-hand column, scaled in size to the rest of the panels. It is clear that the structures seen in the confocal images appear to extend further than the X-ray fluorescence or X-ray phase contrast images. However, the three-dimensional scans show these structures to stand out above the surface only in the same central area of the X-ray images, while the borders are less tall. The two intermediate features in the middle of Fig. 7 ▸ are also lower in thickness; these do not show optical fluorescence in Fig. 1 ▸, so do not contain DNA; they may represent burst nuclei which have lost their DNA.

## 4. Conclusions   

For the human lymphocyte cell nuclei presented in this study, the distributions of P and S, measured by X-ray fluorescence microscopy and associated with the DNA-protein complex of chromatin, are found to be relatively uniform in some examples, such as Figs. 2 ▸ and 4(*b*) ▸, and more strongly modulated in others, such as Figs. 4(*a*) and 4(*c*) ▸. The modulated structure in the P signals of Figs. 4(*a*) and 4(*c*) ▸ resembles the expected pattern of condensed metaphase chromosomes, even though they are not fully resolved. If so, these nuclei are in prophase, since they appear to still possess their nuclear membranes. The more uniform images of Figs. 2 ▸ and 4(*b*) ▸ could be because those cells were in interphase (G1, S, or G2 of the cell cycle), when the chromatin is decondensed, but we note that this is inconsistent with the results in Fig. 5 ▸ which suggest that these two samples contain more DNA than the nuclei of Figs. 4(*a*) and 4(*c*) ▸.

The levels of both P and S vary significantly from nucleus to nucleus, by a factor of two and four, respectively, following the general trend among the samples shown in Fig. 5 ▸. The average level of P is close to the expected values from the number of P atoms contained in the DNA of the human genome in G1 phase, but does not allow a reliable determination of the phase of the cell cycle within current statistics. Both the levels of S and the S/P ratios are found to be higher than expected from the histone proteins alone, which comprise 71% of the total chromosomal protein. This suggests that the non-histone proteins may be richer in Cys and Met residues or that additional proteins are present.

The Fe atom content, while two orders of magnitude lower than P or S, is much more varied among the samples examined, by 15-fold among the integrated signals in Table 1 ▸. Fe is not expected to be associated with DNA in general for evolutionary reasons (Yagi *et al.*, 1992 ▸), yet some exceptions, particularly during DNA replication in S phase, are noted above. Fe is seen to form small bright spots, about 100 nm in diameter, in the samples shown in the low-concentration cases in Figs. 2 ▸ and 4(*b*) ▸. In one case, Fig. 4(*b*) ▸, Fe spots are co-localized with S, perhaps suggesting the presence of FeS enzymes; in the other cases, Fig. 2 ▸, Fe and S are separately localized in spots. There is stronger correlation of Fe with S than with P, suggesting the presence of FeS enzymes, as can also be seen in the overlay plots of Fig. 6 ▸.

High Fe concentration is seen around the edges of the higher-Fe concentration nuclei in Figs. 4(*a*) and 4(*c*) ▸, showing an apparent shell-like structure. Co-localization of Fe and S can be seen especially in the overlay plot of Fig. 6 ▸. These are a strong suggestion of Fe being located in the nuclear membrane, rather than the chromatin-filled centers. In most cases the Fe signal can be seen to surround that of the P and S, suggesting it is associated with the nuclear membrane regions. Since we have less control of the amount of nuclear membrane included in our sample preparation, this may account for the greater variation in the Fe levels than S or P. The relatively low levels of Fe seen in the nuclear interiors may therefore be consistent after all with the evolutionary hypothesis of Yagi *et al.* (1992 ▸).

As far as we can tell, concerning the radiation damage the phase contrast imaging measurements introduced a slight shrinkage (less than 5%) and mass loss (25%) of the nucleus, after one measurement with 0.3 s and seven measurements with longer exposures (1 s). The beam is substantially out of focus here, enlarged to more than the 20 µm × 20 µm field of view in the closest-distance case. However, the raster-scanning SXRF measurement did cause visible changes to the sample; an area shrinkage of about 8% can be found from the comparison of the phase maps and the SXRF maps in Figs. 2 ▸ and 4 ▸. The dose delivered here in SXRF experiments was 3.1 × 10^9^ Gray, which is higher than the dose of 10^7^ Gray known to cause structural changes (Kirz *et al.*, 1995 ▸). However, we observed no elemental mass loss of P from repeated SXRF scans (data not shown).

The post-experiment confocal images, recorded in Fig. 7 ▸, show thinning of the membrane over the entire scanned area, as can be detected in the confocal height map (grey scale image). Multiple overlapping scanned areas can be observed for the upper nucleus, for which the images appear in Fig. 4(*b*) ▸.

## Figures and Tables

**Table 1:** Calibrated X-ray fluorescent signals with their uncertainties, integrated over the five raster scans of human cell nuclei with their DNA preserved Derived masses have been converted into numbers of atoms found within the nuclear regions of the samples measured at ID16A. The nominal thicknesses of nuclei are estimated assuming a density of 1.4 g cm^−3^. The last two rows give the atom counts expected for different phases of the cell cycle, as discussed in the text. The total mass is determined from integration of regions segmented from the phase contrast image.

Sample	P atoms	S atoms	Fe atoms	Thickness (nm)	Ratio P:S	Total mass
Fig. 2 ▸	(1.8 ± 0.1) × 10^10^	(2.6 ± 0.2) × 10^9^	(1.9 ± 0.1) × 10^7^	188 ± 40 nm	6.7	19.7 ± 2.5 pg
Fig. 4(*a*) ▸	(1.3 ± 0.1) × 10^10^	(5.0 ± 0.3) × 10^9^	(3.0 ± 0.2) × 10^8^	490 ± 40 nm	2.6	33.6 ± 2.9 pg
Fig. 4(*b*) ▸	(1.5 ± 0.1) × 10^10^	(4.9 ± 0.3) × 10^9^	(2.4 ± 0.2) × 10^7^	313 ± 40 nm	3.0	27.7 ± 2.2 pg
Fig. 4(*c*) ▸	(1.0 ± 0.1) × 10^10^	(3.5 ± 0.2) × 10^9^	(2.0 ± 0.1) × 10^8^	495 ± 40 nm	2.9	16.3 ± 0.9 pg
Nucleus7	(8.8 ± 0.5) × 10^9^	(1.3 ± 0.1) × 10^9^	(2.0 ± 0.1) × 10^8^	238 ± 40 nm	6.8	17.4 ± 1.0 pg
Average	(1.3 ± 0.2) × 10^10^	(3.5 ± 0.2) × 10^9^	(1.5 ± 0.1) × 10^8^	345 ± 40 nm		22.9 ± 1.9 pg
G1 phase	1.3 × 10^10^	1.0 × 10^9^			13	16.8 pg
G2/M phase	2.6 × 10^10^	2.1 × 10^9^			13	33.6 pg

## References

[bb1] Cloetens, P., Ludwig, W., Baruchel, J., Van Dyck, D., Van Landuyt, J., Guigay, J. P. & Schlenker, M. (1999). *Appl. Phys. Lett.* **75**, 2912–2914.

[bb2] Dawson, J. H. (1988). *Science*, **240**, 433–439. 10.1126/science.3358128 3358128

[bb3] Johnson, D. C., Dean, D. R., Smith, A. D. & Johnson, M. K. (2005). *Annu. Rev. Biochem.* **74**, 247–281. 10.1146/annurev.biochem.74.082803.133518 15952888

[bb4] Kirz, J., Jacobsen, C. & Howells, M. (1995). *Q. Rev. Biophys.* **28**, 33–130. 10.1017/s0033583500003139 7676009

[bb5] Klinge, S., Hirst, J., Maman, J. D., Krude, T. & Pellegrini, L. (2007). *Nat. Struct. Mol. Biol.* **14**, 875–877. 10.1038/nsmb1288 PMC2268749 17704817

[bb6] Kosior, E., Bohic, S., Suhonen, H., Ortega, R., Devès, G., Carmona, A., Marchi, F., Guillet, J. F. & Cloetens, P. (2012). *J. Struct. Biol.* **177**, 239–247. 10.1016/j.jsb.2011.12.005 22182730

[bb7] Lill, R., Dutkiewicz, R., Elsässer, H.-P., Hausmann, A., Netz, D. J. A., Pierik, A. J., Stehling, O., Urzica, E. & Mühlenhoff, U. (2006). *Biochim. Biophys. Acta*, **1763**, 652–667. 10.1016/j.bbamcr.2006.05.011 16843540

[bb8] Mariño-Ramírez, L., Levine, K. M., Morales, M., Zhang, S., Moreland, R. T., Baxevanis, A. D. & Landsman, D. (2011). *Database*, **2011**, bar048. 10.1093/database/bar048 PMC3199919 22025671

[bb9] Morawe, C., Barrett, R., Cloetens, P., Lantelme, B., Peffen, J. C. & Vivo, A. (2015). *Proc. SPIE*, **9588**, 958803.

[bb10] Pacureanu, A., Yang, Y., da Silva, J. C., Baker, R., Barrett, R., Bohic, S., Dabin, Y., Fus, F., Gagliardini, F., Guilloud, C., Hignette, O., Hubert, M., Langer, M., Meyer, J., Morawe, C., Morse, J., Tucoulou-Tachoueres, R., van der Linden, P., Villar, F., Weber, L. & Cloetens, P. (2016). *J. Synchrotron Rad.* In preparation.

[bb11] Robbins, E., Fant, J. & Norton, W. (1972). *Proc. Natl Acad. Sci.* **69**, 3708–3712. 10.1073/pnas.69.12.3708 PMC389854 4509333

[bb12] Roschzttardtz, H., Grillet, L., Isaure, M. P., Conéjéro, G., Ortega, R., Curie, C. & Mari, S. (2011). *J. Biol. Chem.* **286**, 27863–27866. 10.1074/jbc.C111.269720 PMC3151030 21719700

[bb13] Shemilt, L., Verbanis, E., Schwenke, J., Estandarte, A. K., Xiong, G., Harder, R., Parmar, N., Yusuf, M., Zhang, F. & Robinson, I. K. (2015). *Biophys. J.* **108**, 706–713. 10.1016/j.bpj.2014.11.3456 PMC4317545 25650937

[bb14] Solé, V. A., Papillon, E., Cotte, M., Walter, P. & Susini, J. (2007). *At. Spectrosc.* **62**, 63–68.

[bb15] Sukhorukova, E. G., Grigoriev, I. P., Kirik, O. V., Alekseeva, O. S. & Korzhevskii, D. E. (2013). *J. Evol. Biochem. Physiol.* **49**, 370–372.

[bb16] Thompson, K. J., Fried, M. G., Ye, Z., Boyer, P. & Connor, J. R. (2002). *J. Cell Sci.* **115**, 2165–2177. 10.1242/jcs.115.10.2165 11973357

[bb17] Uchiyama, S., Kobayashi, S., Takata, H., Ishihara, T., Hori, N., Higashi, T., Hayashihara, K., Sone, T., Higo, D., Nirasawa, T., Takao, T., Matsunaga, S. & Fukui, K. (2005). *J. Biol. Chem.* **280**, 16994–17004. 10.1074/jbc.M412774200 15687487

[bb18] Wanner, G. & Formanek, H. (1995). *Chromosome Res.* **3**, 368–374. 10.1007/BF00710018 7551552

[bb19] Weiner, B. E., Huang, H., Dattilo, B. M., Nilges, M. J., Fanning, E. & Chazin, W. J. (2007). *J. Biol. Chem.* **282**, 33444–33451. 10.1074/jbc.M705826200 17893144

[bb20] Wu, Y. & Brosh, R. M. (2012). *Nucleic Acids Res.* **40**, 4247–4260. 10.1093/nar/gks039 PMC3378879 22287629

[bb21] Yagi, K., Ishida, N., Komura, S., Ohishi, N., Kusai, M. & Kohno, M. (1992). *Biochem. Biophys. Res. Commun.* **183**, 945–951. 10.1016/s0006-291x(05)80281-5 1314579

[bb22] Yusuf, M., Parmar, N., Bhella, G. K. & Robinson, I. K. (2014). *Biotechniques*, **56**, 257–261. 10.2144/000114168 24806226

